# Snapshot Japan 2023: the first camera trap dataset under a globally standardised protocol in Japan

**DOI:** 10.3897/BDJ.13.e141168

**Published:** 2025-03-13

**Authors:** Keita Fukasawa, Takahiro Morosawa, Yoshihiro Nakashima, Shun Takagi, Takumasa Yokoyama, Masaki Ando, Hayato Iijima, Masayuki U. Saito, Nao Kumada, Kahoko Tochigi, Akira Yoshioka, Satsuki Funatsu, Shinsuke Koike, Hiroyuki Uno, Takaaki Enomoto, William McShea, Roland Kays

**Affiliations:** 1 Biodiversity Division, National Institute for Environmental Studies, Tsukuba, Ibaraki, Japan Biodiversity Division, National Institute for Environmental Studies Tsukuba, Ibaraki Japan; 2 Tokyo University of Agriculture and Technology, Fuchu, Tokyo, Japan Tokyo University of Agriculture and Technology Fuchu, Tokyo Japan; 3 College of Bioresource Science, Nihon University, Fujisawa, Kanagawa, Japan College of Bioresource Science, Nihon University Fujisawa, Kanagawa Japan; 4 University of Hyogo, Tamba,Hyogo, Japan University of Hyogo Tamba,Hyogo Japan; 5 Research Center for Anthropology and Gender Studies, Sugiyama Jogakuen University, Nagoya, Aichi, Japan Research Center for Anthropology and Gender Studies, Sugiyama Jogakuen University Nagoya, Aichi Japan; 6 Faculty of Applied Biological Sciences, Gifu University, Gifu, Gifu, Japan Faculty of Applied Biological Sciences, Gifu University Gifu, Gifu Japan; 7 Forestry and Forest Products Research Institute, Tsukuba, Ibaraki, Japan Forestry and Forest Products Research Institute Tsukuba, Ibaraki Japan; 8 Faculty of Agriculture, Yamagata University, Tsuruoka, Yamagata, Japan Faculty of Agriculture, Yamagata University Tsuruoka, Yamagata Japan; 9 Research Center for Advanced Science and Technology, the University of Tokyo, Meguro, Tokyo, Japan Research Center for Advanced Science and Technology, the University of Tokyo Meguro, Tokyo Japan; 10 Fukushima Regional Collaborative Research Center, National Institute for Environmental Studies, Miharu, Fukushima, Japan Fukushima Regional Collaborative Research Center, National Institute for Environmental Studies Miharu, Fukushima Japan; 11 Graduate School of Natural Science and Technology, Gifu University, Gifu, Gifu, Japan Graduate School of Natural Science and Technology, Gifu University Gifu, Gifu Japan; 12 Graduate School of Agriculture, Tokyo University of Agriculture and Technology, Fuchu, Tokyo, Japan Graduate School of Agriculture, Tokyo University of Agriculture and Technology Fuchu, Tokyo Japan; 13 UGAS, Iwate university, Morioka, Iwate, Japan UGAS, Iwate university Morioka, Iwate Japan; 14 Smithsonian Conservation Biology Institute, Front Royal, United States of America Smithsonian Conservation Biology Institute Front Royal United States of America; 15 North Carolina State University, Raleigh, United States of America North Carolina State University Raleigh United States of America

**Keywords:** camera trap, mammal, bird, Snapshot, biodiversity monitoring, network, East Asia

## Abstract

**Background:**

There is an urgent need to develop global observation networks to quantify biodiversity trends for evaluating achievements of targets of Kunming-Montreal Global Biodiversity Framework. Camera traps are a commonly used tool, with the potential to enhance global observation networks for monitoring wildlife population trends and has the capacity to constitute global observation networks by applying a unified sampling protocol. The Snapshot protocol is simple and easy for camera trapping which is applied in North America and Europe. However, there is no regional camera-trap network with the Snapshot protocol in Asia.

**New information:**

We present the first dataset from a collaborative camera-trap survey using the Snapshot protocol in Japan conducted in 2023. We collected data at 90 locations across nine arrays for a total of 6162 trap-nights of survey effort. The total number of sequences with mammals and birds was 7967, including 20 mammal species and 23 avian species. Apart from humans, wild boar, sika deer and rodents were the most commonly observed taxa on the camera traps, covering 57.9% of all the animal individuals. We provide the dataset with a standard format of Wildlife Insights, but also with Camtrap DP 1.0 format. Our dataset can be used for a part of the global dataset for comparing relative abundances of wildlife and for a baseline of wildlife population trends in Japan. It can also used for training machine-learning models for automatic species identifications.

## Introduction

Monitoring global trends in biodiversity is crucial to understand the impact of climate and land-use change on biodiversity and to develop effective conservation strategy to halt biodiversity loss (Jetz et al. 2019). The mission of Kunming-Montreal Global Biodiversity Framework (KM-GBF) is to take urgent action to halt and reverse biodiversity loss to put nature on a path to recovery (Convention on Biological Diversity 2022). Development of observation networks is an urgent task for evaluating biodiversity trend and achievement of the long-term goals of KM-GBF (Gonzalez et al. 2023). While assessments of international efforts against climate change are based on internationally standardised sensor networks, the lack of standardisation of observation methods in global biodiversity conservation is a major gap for the effective global assessment (Kissling et al. 2018).

Camera trapping is a cost effective measure for wildlife monitoring and has potential to develop a globally standardised sensor network for biodiversity observation (Steenweg et al. 2017). Camera traps are effective for medium- to large-sized mammals and birds, which are especially threatened by global changes resulting from human activities (Harfoot et al. 2021) and also known foci of human-wildlife conflicts such as crop damage and zoonoses (Uchida et al. 2023). Recently, a standardised camera-trapping protocol was adopted to coordinate camera-trapping projects over the world (Cove et al. 2021, Pardo et al. 2021, Kays et al. 2022, Shamon et al. 2024) under the common title of "Snapshot". Although the observation network of Snapshot has a potential for offering valuable information on population trends and behaviour of animal and birds globally, expansion of the spatial coverage is needed.

Japan is located at the easternmost part of Palearctic and Indo-Malay biogeographic realms (Olson et al. 2001) and is a known biodiversity hotspot of the world (Mittermeier et al. 2004). Mammals in Japan maintained high diversity over the Holocene period due to the limited development of ice sheets during the last glacial period and the long-term land-use history would affect the faunal patterns in Japan (Fukasawa and Akasaka 2019, Iijima et al. 2023). The recent socio-economic change towards depopulation of rural areas and underuse of wildlife resources has resulted in the range expansions of some mammalian species (Ohashi et al. 2016, Saito et al. 2016) and increase in human-wildlife conflict (Tsunoda and Enari 2020). Although there is an urgent need for monitoring data, such as wildlife abundance indices, being made available to the public, there are limited open datasets in Japan (Biodiversity Center of Japan 2015, Fukasawa et al. 2016, Enari and Enari 2020). Moreover, previous open datasets did not include camera-trap images, limiting their applicability to train machine-learning models for species identifications.

This data paper provides a dataset of mammal and bird communities in Japan in 2023 obtained from the first collaborative camera-trap survey under the standardised protocol of Snapshot. This project involved 15 scientists setting camera traps at 90 locations across nine arrays for a total of 6162 trap-nights of survey effort. We provide the dataset with a standard format of Wildlife Insights (Wildlife Insights 2020), but also with Camtrap DP 1.0 format (Bubnicki et al. 2023). Images will also be available in Wildlife Insights upon acceptance of the data paper, which will be the first open camera trap-images in Japan. Our dataset can be used for global-scale studies by integrating it with Snapshot protocol camera-trap data from other regions of the world. Our intent is to expand the survey to emcompass more regions and collaborators. It can also be used for baseline data of future trend assessments of biodiversity and for training machine-learning models for species identifications.

## General description

### Purpose

As with Snapshot USA (Cove et al. 2021, Kays et al. 2022), the purpose of Snapshot Japan is to facilitate collaboration in creating a national database of public wildlife data. These data will be provided annually for use in conservation and ecological research to examine regional and nationwide trends in mammal communities associated with environmental and anthropogenic landscape variables. This information will help to better inform wildlife management and conservation actions.

## Project description

### Study area description

The survey was conducted on Honshu Island and two accompanying small islands (Awaji Island and Omishima Island) in Japan. Mean annual temperature and annual precipitation in the capital city, Tokyo, are 15.8ºC and 1598.2mm/m^2^, respectively (Japan Meteorological Agency 2021). Our study covers the three largest ecoregions in Honshu Island, Nihonkai montane deciduous forests, Taiheiyo evergreen forests and Taiheiyo montane deciduous forests (Olson et al. 2001). Our data cover wild forest habitat in the rural and natural landscape, as well as planted forests and agricultural lands.

## Sampling methods

### Study extent

**Figure 1.**
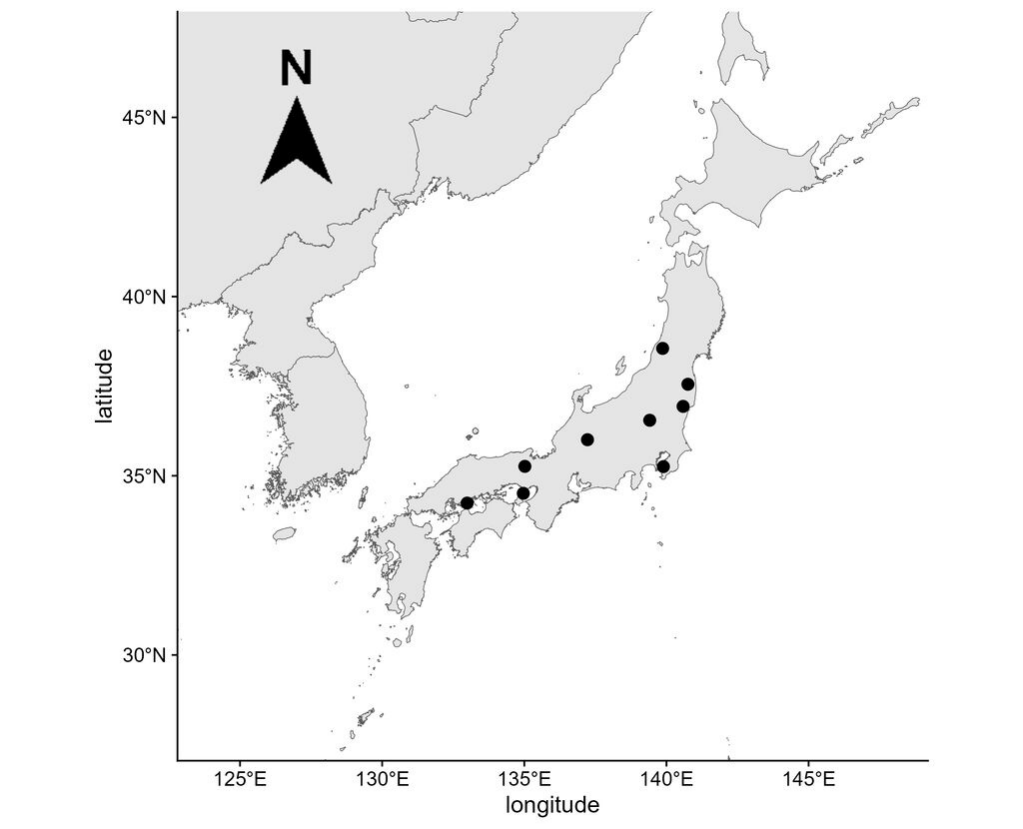
Locations of camera-trapping arrays. A black dot indicates the centroid of camera-trap locations in an array.

We conducted camera-trap surveys at 90 locations across nine arrays (clusters of camera trap sites) in eight Prefectures (i.e. the major governmental subdivisions of Japan) (Fig. 1). The latitudinal and longitudinal range of camera-trap locations are from 34.21N to 38.65N and from 132.97E to 140.82E, respectively. The number of camera-trap locations within each array varied between 9 and 12 (mean: 10.0) depending on logistics and equipment. Distance from a camera trap location to the nearest neighbour within an array was required to be within the range of 200 m and 5 km. We installed a camera trap in each location and operated in the survey period of September and October, producing 550-1060 camera-nights with a mean of 685 camera-nights of survey effort per array.

### Sampling description

Our sampling protocol is Snapshot USA (Cove et al. 2021, Kays et al. 2022) compliant. We coordinated surveys with all research groups with the goal of collecting a minimum of 400 camera-nights of camera-trap data from camera-trap arrays during the months of September and October 2023, but the data obtained beyond these time periods are included. As long as the deployment in the dataset is properly defined, interruptions due to implementation limitation in the field are not prohibited. However, limiting daily active times by camera-trap settings was not allowed. Cooperators adhered to the following protocol:

1. Each research group self-identified their camera trap arrays by selecting one combination of setting (Urban, Suburban, Rural, Wild) and habitat (Forest, Grassland, Desert, Anthropogenic, Other) to standardise and focus on where deploying their camera-trap sites in each array, although our data included only the three combinations, Rural-Forest, Wild-Forest and Rural-Anthroponegic. Camera-trap arrays were amorphous and not necessarily systematic grids, with random sites selected to fit within study spacing guidelines and logistical constraints of a particular location. Camera spacing guidelines allowed for sites that were at least 200 m apart from other cameras, with no single cameras greater than 5 km from another camera.
2. Camera traps were deployed across more than eight camera-trap sites per a camera-trap array. However, due to camera functional issues, the effective number of camera-trap sites in each array ranged from 8 to 12 cameras (mean: 9.78). The location, start date, end date, camera brand and model and functional status of the camera trap for each deployment were recorded. We ensured that all cooperators used comparable motion-sensitive, infrared or white-flash trail cameras with fast (< 0.5 s) trigger speeds. Cameras were set to take 1-5 photos per trigger and cameras were set up to take photos without a quiet period between triggers. All the cameras deployed were with a trigger time < 0.5 seconds including Browning (Strike Force HD Pro X, Strike Force Pro X 1080 and Dark Ops Apex), Bushnell (Trophycam 24MT, Trophy Cam HD Aggressor No glow, Core 24MP No Glow and Core S-4K No Glow 30MP) and Stealth Cam (STC-G42NG).
3. Cameras were placed ~ 50 cm off the ground, parallel to the ground with horizontal orientation. The direction of each camera was not standardised or recorded, but we suggested that camera orientation to the north or south is effective to reduce sun glare in resulting camera trap images.
4. No food bait or scent lure was used.
5. Cameras could be placed on trails or logging roads or water features, but this was indicated by operators.

After removing camera traps, project operators uploaded all data to Wildlife Insights (https://www.wildlifeinsights.org/). Wildlife Insights aggregates photos into sequences such that species detections were only considered independent if they were greater than one-minute apart. Each participant identified the species in their sequences using the Wildlife Insights to register this information.

### Quality control

We followed the Snapshot USA procedure for quality control (Cove et al. 2021, Kays et al. 2022). Species identifications were then reviewed by another expert to confirm or correct all species identifications or counts. We rejected any deployments that did not meet standard procedures, which were typically deployed too high or too low to detect most common species. Three authors (Y. Nakashima, T. Morosawa, K. Fukasawa) confirmed all difficult species identifications and were responsible for most of the expert review of Snapshot Japan deployments in Wildlife Insights. We reached out to taxonomical experts when necessary and adjusted species identifications to the lowest possible taxonomical unit when unable to identify an animal to species. Non-target species (e.g. reptiles and insects) were typically only identified to class because species identification was difficult, but also because camera traps are not reliable survey approaches for these species in the current study design.

To ensure the capacity to integrate our dataset with other regions of the world, we adopted a standard taxonomy of Wildlife Insights, the combination of IUCN Red List of Endangered Species (IUCN 2024) and the American Society of Mammalogists Mammal Diversity Database for mammals (Burgin et al. 2018) and Birdlife International's taxonomy for birds (HBW and BirdLife International 2024).

### Step description

As described in the section **Sampling description**, image data was uploaded to Wildlife Insights after retrieval from the field and the images taken continuously with a time lag of less than 1 minute were grouped into a sequence using the Wildlife Insights function. After that, bounding boxes of animals and humans were embedded on images automatically with a pre-trained AI model of Wildlife Insights. After tagging the species found and the group size for all the sequences (including sequences without bounding boxes) manually, independent researchers reviewed all the records and corrected the erroneous identifications (section **Quality control**). The dataset of detections and deployments was exported from Wildlife Insights. Images are available publicly on Wildlife Insights following publication. Human images were deleted from database for protection of privacy.

## Geographic coverage

### Description

Honshu Island and two accompanying islands (Awaji Island and Omishima Island) in Japan.

### Coordinates

34.21143N and 38.56029N Latitude; 140.8155E and 132.9668E Longitude.

## Taxonomic coverage

### Description

Mammals and birds were identified to species levels where possible. Sequences that could not be discriminated to the species level were only classified to genus or higher levels. Non-target species such as reptiles and insects were identified to class level.

### Taxa included

**Table:** 

Rank	Scientific Name	Common Name
class	Mammalia	mammal
class	Aves	bird

## Temporal coverage

### Notes

August 2023 to December 2023 (mainly September and October 2023), for a total of 6162 trap-nights of survey effort.

## Usage licence

### Usage licence

Open Data Commons Attribution License

## Data resources

### Data package title

Snapshot Japan 2023

### Number of data sets

3

### Data set 1.

#### Data set name

deployments.csv

#### Data format

Wildlife Insights deployments

#### Description

The deployment dataset was published via Wildlife Insights (2020). It was formatted with .csv including locations, start and end dates and unique identifiers of camera trap deployments (Suppl. material 1). It contains the 90 camera-trap deployments obtained through Snapshot Japan 2023. In addition, the dataset formatted by camtrap DP 1.0 was supplied in Supporting Information.

**Data set 1.** 

Column label	Column description
project_id	Unique identifier of projects in Wildlife Insights. Seven-digit integer.
deployment_id	Unique identifier of camera deployments. Character string.
placename	Name of camera trap location. Character string.
latitude	Latitude in decimal notation. Double-precision floating point number.
longitude	Longitude in decimal notation. Double-precision floating point number.
start_date	Dates and times when camera traps were activated. The time zone is Japan Standard Time. Formatted to "yyyy-mm-dd hh:mm:ss".
end_date	Dates and times when camera traps were stopped. The time zone is Japan Standard Time. Formatted to "yyyy-mm-dd hh:mm:ss".
bait_type	Type of bait used. There were only "None" in our dataset.
bait_description	Detailed description of bait. All the records in our data are blank.
feature_type	Feature types of objects surveyed by camera traps (e.g. road and trails). Character string.
feature_type_methodology	Text field to describe the feature type methodology. Unused in our dataset.
camera_id	Unique identifiers of camera traps used. They were given by Wildlife Insights. Seven-digit integer.
camera_name	Names of camera traps. Character string.
quiet_period	Time specified between shutter triggers when activity in the sensor will not trigger the shutter. Values were rounded to seconds.
camera_functioning	Camera functioning status (e.g. Wildlife Damage).
sensor_height	Height of camera traps from the ground. Only "knee height" in our dataset.
height_other	Detail description of camera height. Unused column in our dataset.
sensor_orientation	Tilt angle of camera trap. Our dataset only includes "Parallel", indicating the angle of a camera trap is parallel to ground.
orientation_other	Detail description of sensor_orientation. Unused column in our dataset.
plot_treatment	A parcel of land defined by a specific function, property or purpose. Examples include types of agriculture and different stages of controlled burns. Unused column in our dataset.
plot_treatment_description	General description of the plot treatment. Unused column in our dataset.
detection_distance	Maximum distance at which a camera triggered, as tested during deployment, measured in metres. Unused column in our dataset.
subproject_name	Names of camera trap arrays. Character string.
subproject_design	Aims and survey designs of camera trap arrays. Unused in our dataset.
event_name	Names of sampling event groups such as seasons, months, years or other types of logical groupings. Unused column in our dataset.
event_description	A description that defines the events. Unused column in our dataset.
event_type	A broader category for types of events. Unused column in our dataset.
recorded_by	The person installing the camera. Unused column in our dataset.
remarks	Any other note about the deployment.

### Data set 2.

#### Data set name

sequences.csv

#### Data format

Wildlife Insights sequencess

#### Description

The observations dataset was published via Wildlife Insights (2020). It was formatted with .csv including dates, times, species, group sizes and deployment identifiers of sequences (Suppl. material 2). The deployment identifiers are common keys to deployments dataset. It contains the observations of 18027 sequences (including blanks and detections of non-target taxa) obtained through Snapshot Japan 2023. In addition, the dataset formatted by camtrap DP 1.0.1 was also supplied (Suppl. material 4) and will be published through GBIF (https://doi.org/10.15468/y4erss).

**Data set 2.** 

Column label	Column description
project_id	Unique identifiers of projects in Wildlife Insights. Seven-digit integer.
deployment_id	Unique identifier of camera deployments. Character string.
sequence_id	Unique idenitifiers of sequences. Seven-digit integer.
is_blank	Indicator values whether the sequences are blank (1) or not (0).
identified_by	Name of person who identified the species in a sequence. Character string.
wi_taxon_id	Identifiers of taxa in the Wildlife Insights checklist. Specified as Universally Unique Identifier (UUID).
class	Class of animals.
order	Order of animals.
family	Family of animals.
genus	Genus of animals.
species	Species of animals.
common_name	Common name of animals.
uncertainty	Uncertainty levels of identifications. Unused column in our dataset.
start_time	Start times of sequences. Formatted to "yyyy-mm-dd hh:mm:ss".
end_time	End times of sequences. Formatted to "yyyy-mm-dd hh:mm:ss".
group_size	Group size of animals in a sequence.
age	Optional values indicating adult or juvenile of detected individuals. Blank indicates the age has not been examined and the value 'unknown' means the age has been examined, but is unidentifiable.
sex	Optional values indicating the sex of detected individuals.
animal_recognisable	This column was generated by Wildlife Insights, but unused in our dataset.
individual_id	Identifiers of tagged individuals. Unused column in our dataset.
individual_animal_notes	Notes about sequences and the detected individuals. Character string.
behaviour	Optional description of behaviour of an individual (e.g. eating).
highlighted	Tags of individuals which were highlighted in Wildlife Insights. Unused column in our dataset.
markings	This column was generated by Wildlife Insights, but unused in our dataset.
cv_confidence	This column was generated by Wildlife Insights, but unused in our dataset.
licence	Licence of images uploaded to Wildlife Insights. In our study, the licence of images are the Creative Commons Attribution 4.0 International license ('CC BY'), the full text of which is available at https://creativecommons.org/licenses/by/4.0/legalcode.

### Data set 3.

#### Data set name

habitats.csv

#### Description

This data table describes the prefectures, habitats, landscape settings and ecoregions of subprojects (Suppl. material 3). To determine the ecoregions of subprojects, we used the polygon dataset by Dinerstein et al. (2017).

**Data set 3.** 

Column label	Column description
subproject_name	Names of camera-trap arrays. Character string.
prefecture	Prefectures of the camera-trap arrays. Character string.
habitat_type	Habitat types of the camera-trap arrays. Character string.
landscape_type	Landscape settings of the camera-trap arrays. Character string.
ECO_NAME	Names of ecoregions of subprojects. Character string.

## Additional information

**Results**

Total number of sequences in which mammals and birds were detected was 7967 and consist of 386 sequences with avian species and 7582 sequences with mammalian species (i.e. a sequence with both). Multiple individuals were detected in 12.15% (968 out of 7967) of sequences and the total number of individual detections was 9414 (425 birds and 8989 mammals). The data include 20 mammalian species and 23 avian species (Table 1). The seven non-native species, northern raccoon (*Procyonlotor*), domestic goat (*Capraaegagrushicus*), domestic cat (*Feliscattus*), masked palm civet (*Pagumalarvata*), domestic dog (*Canisfamilaris*), Chinese bamboo partridge (*Bambusicolathoracicus*) and Hwamei (*Garrulaxcanorus*) was detected. The top three dominant non-human mammals, wild boar (*Susscrofa*), sika deer (*Cervusnippon*) and rodent (order Rodentia), covered 57.9% of all the animal detections.

**Figure 2.**
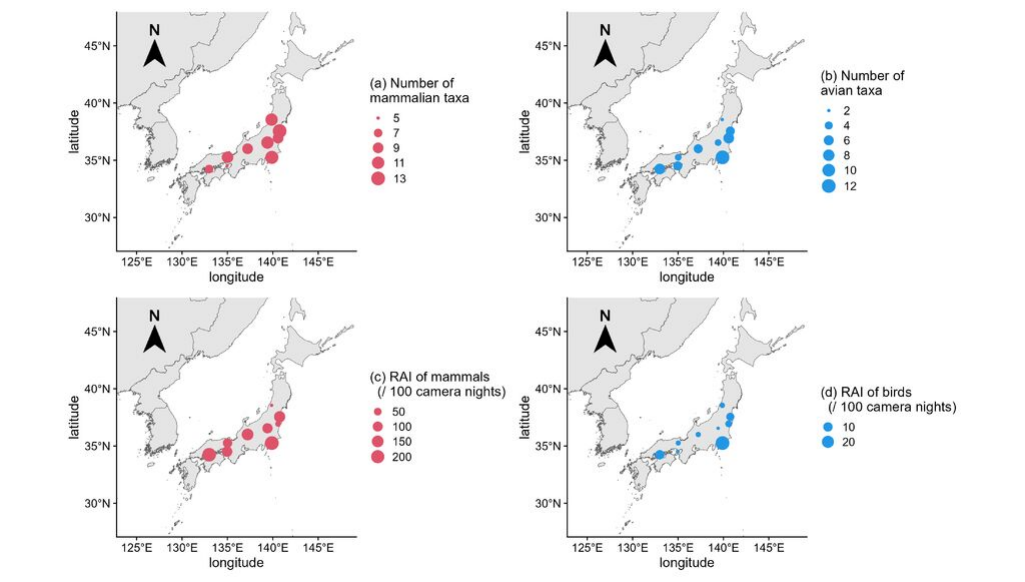
Map of the number of taxa for (a) mammals and (b) birds and RAI of (c) mammals and (d) birds for each camera-trap array.

The numbers of taxa and relative abundance indices (RAIs, O’Brien 2011) varied amongst the camera-trap arrays (Fig. 2). The number of mammalian and avian taxa ranged from 5 to 13 and 2 to 12, respectively. The smaller island arrays tended to have smaller numbers of mammalian taxa: five taxa for the array of Hyogo_Forest_Awaji370 (Awaji Island, 592.55 km^2^) and seven taxa for the array of Ehime_Artificial_Omishima (Omishima Island, 64.54 km^2^) (Fig. 2a), whereas the number of avian taxa did not show such a clear trend (Fig. 2b). The ranges of RAIs (per 100 trap nights) were [14.4, 223] for mammals and [0.974, 28.1] for birds and the array of Chiba was the highest for both mammals and birds (Fig. 2cd).

**Figure 3.**
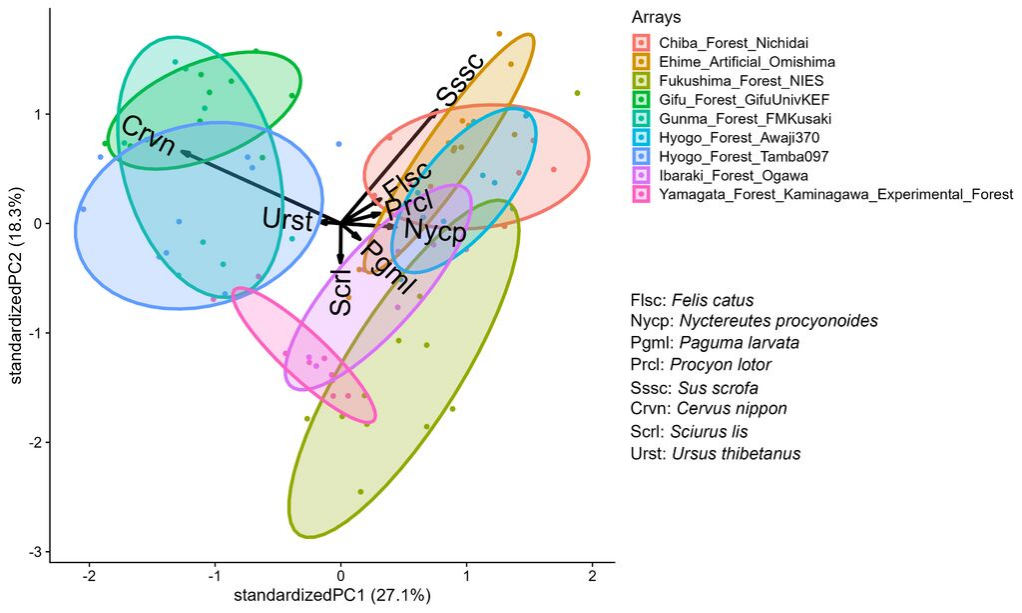
Biplot of principal component analysis for the relative abundance indices of the mammal community. The ellipsoids correspond to camera-trap arrays. The eight longest vectors of species are shown as arrows.

**Figure 4.**
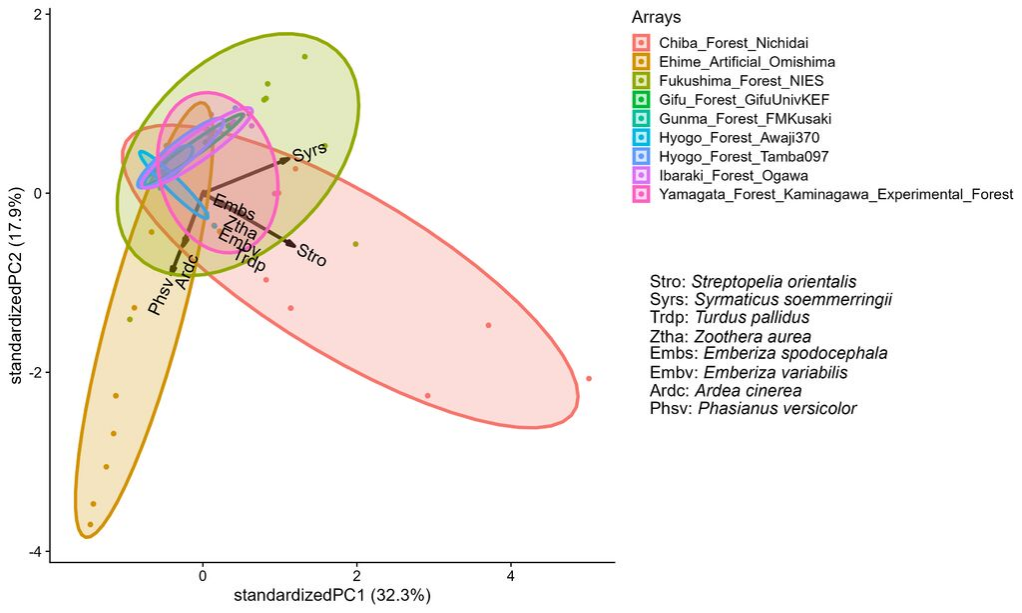
Biplot of principal component analysis for the relative abundance indices of the avian community. The ellipsoids correspond to camera-trap arrays. The eight longest vectors of species are shown as arrows.

To summarise the community structure detected, we conducted principal component analyses (PCAs) for both mammals and birds. For the PCAs, we used RAIs as the input variables. We included detections identified to species only in the analysis. To stabilise the variance of input variables, we ln(x + 1)-transformed RAIs. The outputs of PCAs are principal component scores of sample locations and the first two principal components (PC1 and PC2) correspond to a projection of community compositions in a multidimensional space on to a two dimensional plane with the largest variances. The 45.4% and 50.3% of variances were explained by the first two principal components (PCs) for mammals and birds, respectively (Figs 3, 4). The results indicated that compositions of mammal species were similar amongst locations in the same camera-trap array and locations in each camera-trap array showed clumped distribution in the principal component space (Fig. 3). Sika deer were detected in the five arrays and associated strongly with PC1 with a correlation coefficient of -0.84. Wild boar were detected in all the arrays, but heterogeneity in RAIs were large enough to characterise the first two principle axes (PC1 and PC2) with correlation coefficients 0.57 and 0.77, respectively. Non-native species (domestic cat, masked palm civet and northern raccoon) were positively associated with PC1. Contrary to the mammals, intra-array variations were dominant in the compositions of avian communities (Fig. 4). Vectors of multiple species were overlaped, reflecting the co-occurrences of many species at a small number of camera-trap locations.

**Discussion**

To evaluate biodiversity trends over the world, standardised observation which allows us to integrate and compare with datasets obtained from different places and times is essential (Steenweg et al. 2017, Gonzalez et al. 2023). To date, collaborative camera-trap monitoring initiatives were launched over the world (Jansen et al. 2014, Cove et al. 2021, Pardo et al. 2021, Kays et al. 2022), but any camera-trapping initiative with a globally-standardised protocol does not cover the temperate East Asian region to our knowledge. We took a collaborative approach for camera-trap monitoring and obtained a dataset which covered a large geographical extent. This dataset offered not only baselines for wildlife population trends in Japan, but also a proof-of-concept for feasibility of collaborative Snapshot protocol in eastern Asia.

In Japan, overabundance of wildlife due to underuse poses threats to human well-being and biodiversity (Tsunoda and Enari 2020). Our dataset showed the dominance of human-wildlife conflict agents. Two of the species most commonly implicated in human-wildlife conflict in Japan, sika deer and wild boar, were also the most often detected according to our dataset (Table 1). However, their dominance are heterogeneous amongst arrays (Fig. 3). In addition to land use and climate factors (Saito et al. 2016, Ohashi et al. 2016), epidemiological factors can also play a role in the heterogeneous patterns. In 2018, a classical swine fever (CSF) outbreak occurred in Japan and continues to expand its range in 2023 (Sawai et al. 2021). Population density of wild boar decreased immediately after CSF invasion (Ikeda et al. 2021) and recovered as the antibodies spread throughout the population (Shimizu et al. 2021). Thus, RAIs of wild boar may depend on the timing of CSF invasion. For example, the array "Fukushima_Forest_NIES" belongs to the invasion front of CSF and the outbreak is ongoing (World Organisation for Animal Health 2021). The camera trap locations of this array were plotted at the opposite side of the wild boar vector on the major PC space (Fig. 3), implying the impact of CSF outbreak. Monitoring the transient dynamics of wildlife with camera traps will contribute to understand community-level response of wildlife to infectious disease. Invasive alien species (IAS) are a major threat to island ecosystems (Clavero and Garciaberthou 2005) and our data showed non-native species were a major part of the mammalian community in Japan (Table 1). Collaborative camera-trap monitoring will contribute to evaluate the risk of increase of IAS and the effectiveness of control measures.

The potential of our dataset to address ecological and management issues will improve further by integrating other datasets. Intercontinental comparisons between Snapshot initiatives (e.g. Swanson et al. (2015), Cove et al. (2021), Kays et al. (2022)) will offer insights on general rules of community assemblage of terrestrial animals. Integrative analysis of our dataset and species occurrence data including the citizen-science dataset, such as iNaturalist (2008), will contribute to improve the performance of species distribution modelling (Koshkina et al. 2017). For evaluations of wildlife and IAS management, integration with hunting records will be valuable because our dataset offers reliable RAIs which are required to estimate hunting mortalities and population size trends correctly using harvest-based models (Fukasawa et al. 2020). Calibration of RAIs by independent absolute population density estimates by unmarked abundance estimators, such as random encounter and staying time model (Nakashima et al. 2018) and camera-trap distance sampling (Howe et al. 2017) will improve reliablity of population trend and broaden the applicability of RAIs to predict absolute population density.

Non-uniform sampling intensities over taxa and space are quite common in large-scale datasets and our dataset is not an exception. Although our survey methods are capable of detecting mammals and birds within sight of the camera traps, detection of arboreal, subterranean and flying species would be incomplete and involve great uncertainty. If it is worth the extra implementation cost, combined use of arboreal (Suzuki and Ando 2018) and subterranean camera traps (Soininen et al. 2015) and acoustic monitoring tools (Fukasawa et al. 2017, Hoggatt et al. 2024) may be beneficial in improving coverage of taxa. Considering the arbitrariness of camera-trap array selection that is unavoidable in collaborative monitorings, it is advisable for users to be aware of the distributional bias of arrays in geographical coordinates, climate and land use and to apply statistical methods, robust against sampling bias (Horvitz and Thompson 1952, Isaac et al. 2020).

In conclusion, our initiative will strengthen the global camera-trap monitoring network by filling the gap in the East Asian region. We will continue the monitoring in the next year and thereafter for evaluation of wildlife trends, which will offer improved indicators for KM-GBF targets. Recruiting more sites both in Japan and the region to cover more broad climatic regions and land use is an urgent task. We call for international collaborations on the standardised camera-trap network with other initiatives in Asian countries.

## Supplementary Material

77D675BA-E4B7-5639-B320-E3392AB8DC4D 10.3897/BDJ.13.e141168.suppl1 Supplementary material 1 deployments.csv Data type deployments File: oo_1202827.csv https://binary.pensoft.net/file/1202827 Keita Fukasawa, Takahiro Morosawa, Yoshihiro Nakashima, Shun Takagi, Takumasa Yokoyama, Masaki Ando, Hayato Iijima, Masayuki Saito, Nao Kumada, Kahoko Tochigi, Akira Yoshioka, Satsuki Funatsu, Shinsuke Koike, Hiroyuki Uno, Takaaki Enomoto

3184C9A5-DE92-5429-AE61-2D0D8B67A85E 10.3897/BDJ.13.e141168.suppl2 Supplementary material 2 sequences.csv Data type occurrences File: oo_1165478.csv https://binary.pensoft.net/file/1165478 Keita Fukasawa, Takahiro Morosawa, Yoshihiro Nakashima, Shun Takagi, Takumasa Yokoyama, Masaki Ando, Hayato Iijima, Masayuki Saito, Nao Kumada, Kahoko Tochigi, Akira Yoshioka, Satsuki Funatsu, Shinsuke Koike, Hiroyuki Uno, Takaaki Enomoto

35DEC245-DB2A-5E6A-AFCB-7EA16463B231 10.3897/BDJ.13.e141168.suppl3 Supplementary material 3 habitats.csv Data type habitat and landscape category of subprojects File: oo_1203756.csv https://binary.pensoft.net/file/1203756 Keita Fukasawa, Takahiro Morosawa, Yoshihiro Nakashima, Shun Takagi, Takumasa Yokoyama, Masaki Ando, Hayato Iijima, Masayuki Saito, Nao Kumada, Kahoko Tochigi, Akira Yoshioka, Satsuki Funatsu, Shinsuke Koike, Hiroyuki Uno, Takaaki Enomoto

2FA05BB2-729A-5005-AF5A-D203751FFC4C 10.3897/BDJ.13.e141168.suppl4 Supplementary material 4 Dataset formatted as Camtrap DP 1.0.1 Data type Data package File: oo_1246258.zip https://binary.pensoft.net/file/1246258 Keita Fukasawa, Takahiro Morosawa, Yoshihiro Nakashima, Shun Takagi, Takumasa Yokoyama, Masaki Ando, Hayato Iijima, Masayuki Saito, Nao Kumada, Kahoko Tochigi, Akira Yoshioka, Satsuki Funatsu, Shinsuke Koike, Hiroyuki Uno, Takaaki Enomoto

## Figures and Tables

**Table 1.** Number of individuals of mammals and birds detected and the number of arrays and locations where the species were detected. Species with * are non-natives in Japan. The IUCN Red List ranks were also shown. Note that there was no species designated as the national Red List by the Ministry of Environment of Japan.

Common name	Class	Order	Family	Genus	Species	No. of individual detections	Number of arrays	Number of locations	Non-native species	IUCN Red List rank
Wild Boar	Mammalia	Cetartiodactyla	Suidae	*Sus*	*scrofa*	2580	9	67		LC
Sika Deer	Mammalia	Cetartiodactyla	Cervidae	*Cervus*	*nippon*	1766	5	35		LC
Human	Mammalia	Primates	Hominidae	*Homo*	*sapiens*	872	9	89		
Rodent	Mammalia	Rodentia				602	8	28		
Raccoon Dog	Mammalia	Carnivora	Canidae	*Nyctereutes*	*procyonoides*	587	9	58		LC
Mammal	Mammalia					314	8	53		
Northern Raccoon	Mammalia	Carnivora	Procyonidae	*Procyon*	*lotor*	305	2	12	*	LC
Japanese Squirrel	Mammalia	Rodentia	Sciuridae	*Sciurus*	*lis*	269	4	16		LC
Domestic Goat	Mammalia	Cetartiodactyla	Bovidae	*Capra*	*aegagrus hircus*	265	1	1	*	
Reeves' Muntjac	Mammalia	Cetartiodactyla	Cervidae	*Muntiacus*	*reevesi*	245	1	6	*	LC
Weasel Family	Mammalia	Carnivora	Mustelidae			170	6	26		
Japanese Hare	Mammalia	Lagomorpha	Leporidae	*Lepus*	*brachyurus*	169	6	20		LC
Japanese Badger	Mammalia	Carnivora	Mustelidae	*Meles*	*anakuma*	147	6	21		LC
Japanese Macaque	Mammalia	Primates	Cercopithecidae	*Macaca*	*fuscata*	139	4	16		LC
Domestic Cat	Mammalia	Carnivora	Felidae	*Felis*	*catus*	96	7	27	*	
Oriental Turtle-dove	Aves	Columbiformes	Columbidae	*Streptopelia*	*orientalis*	90	5	15		LC
Japanese Marten	Mammalia	Carnivora	Mustelidae	*Martes*	*melampus*	87	7	30		LC
Copper Pheasant	Aves	Galliformes	Phasianidae	*Syrmaticus*	*soemmerringii*	81	7	31		NT
Small Mammal	Mammalia					74	4	15		
Masked Palm Civet	Mammalia	Carnivora	Viverridae	*Paguma*	*larvata*	73	7	23	*	LC
Japanese Serow	Mammalia	Cetartiodactyla	Bovidae	*Capricornis*	*crispus*	64	4	19		LC
Bird	Aves					56	6	19		
Carnivorous Mammal	Mammalia	Carnivora				39	4	19		
Green Pheasant	Aves	Galliformes	Phasianidae	*Phasianus*	*versicolor*	34	2	7		LC
Red Fox	Mammalia	Carnivora	Canidae	*Vulpes*	*vulpes*	33	5	10		LC
Asiatic Black Bear	Mammalia	Carnivora	Ursidae	*Ursus*	*thibetanus*	29	4	17		VU
Jay	Aves	Passeriformes	Corvidae	*Garrulus*	*glandarius*	26	6	15		LC
Grey Heron	Aves	Pelecaniformes	Ardeidae	*Ardea*	*cinerea*	23	1	4		LC
Pale Thrush	Aves	Passeriformes	Turdidae	*Turdus*	*pallidus*	22	3	6		LC
Domestic Dog	Mammalia	Carnivora	Canidae	*Canis*	*familiaris*	18	2	5	*	
Japanese Weasel	Mammalia	Carnivora	Mustelidae	*Mustela*	*itatsi*	17	3	10		NT
Bat	Mammalia	Chiroptera				16	5	12		
Chinese Bamboo Partridge	Aves	Galliformes	Phasianidae	*Bambusicola*	*thoracicus*	10	1	2	*	LC
White's Thrush	Aves	Passeriformes	Turdidae	*Zoothera*	*aurea*	9	2	5		LC
Grey Bunting	Aves	Passeriformes	Emberizidae	*Emberiza*	*variabilis*	8	1	2		LC
Eastern Great Tit	Aves	Passeriformes	Paridae	*Parus*	*minor*	8	2	3		LC
Turdus Species	Aves	Passeriformes	Turdidae	*Turdus*		7	2	5		
Passeriformes Order	Aves	Passeriformes				6	1	2		
Weasel Species	Mammalia	Carnivora	Mustelidae	*Mustela*		5	2	3		
Phasianidae Family	Aves	Galliformes	Phasianidae			5	5	5		
Japanese Thrush	Aves	Passeriformes	Turdidae	*Turdus*	*cardis*	5	2	4		LC
Apodemus Species	Mammalia	Rodentia	Muridae	*Apodemus*		4	1	1		
Meadow Bunting	Aves	Passeriformes	Emberizidae	*Emberiza*	*cioides*	4	2	2		LC
Brown-eared Bulbul	Aves	Passeriformes	Pycnonotidae	*Hypsipetes*	*amaurotis*	4	2	4		LC
Eyebrowed Thrush	Aves	Passeriformes	Turdidae	*Turdus*	*obscurus*	4	2	2		LC
Brown-headed Thrush	Aves	Passeriformes	Turdidae	*Turdus*	*chrysolaus*	3	2	3		LC
Emberiza Species	Aves	Passeriformes	Emberizidae	*Emberiza*		2	1	1		
Japanese Wagtail	Aves	Passeriformes	Motacillidae	*Motacilla*	*grandis*	2	1	1		LC
Great White Egret	Aves	Pelecaniformes	Ardeidae	*Ardea*	*alba*	2	1	1		LC
Japanese Night-heron	Aves	Pelecaniformes	Ardeidae	*Gorsachius*	*goisagi*	2	1	1		VU
Japanese Woodpecker	Aves	Piciformes	Picidae	*Picus*	*awokera*	2	2	2		LC
Cervidae Family	Mammalia	Cetartiodactyla	Cervidae			1	1	1		
Cetartiodactyla Order	Mammalia	Cetartiodactyla				1	1	1		
Cricetidae Family	Mammalia	Rodentia	Cricetidae			1	1	1		
Japanese Dormouse	Mammalia	Rodentia	Gliridae	*Glirulus*	*japonicus*	1	1	1		LC
Columbidae Family	Aves	Columbiformes	Columbidae			1	1	1		
Corvus Species	Aves	Passeriformes	Corvidae	*Corvus*		1	1	1		
Black-faced bunting	Aves	Passeriformes	Emberizidae	*Emberiza*	*spodocephala*	1	1	1		LC
Hwamei	Aves	Passeriformes	Leiotrichidae	*Garrulax*	*canorus*	1	1	1	*	LC
Garrulax Species	Aves	Passeriformes	Leiotrichidae	*Garrulax*		1	1	1		
Narcissus Flycatcher	Aves	Passeriformes	Muscicapidae	*Ficedula*	*narcissina*	1	1	1		LC
Muscicapidae Family	Aves	Passeriformes	Muscicapidae			1	1	1		
Varied tit	Aves	Passeriformes	Paridae	*Sittiparus*	*varius*	1	1	1		LC
Dendrocopos Species	Aves	Piciformes	Picidae	*Dendrocopos*		1	1	1		
Otus Species	Aves	Strigiformes	Strigidae	*Otus*		1	1	1		
